# Magnetic Resonance Imaging versus Electrophysiologic Tests in Clinical Diagnosis of Lower Extremity Radicular Pain

**DOI:** 10.1155/2013/952570

**Published:** 2013-01-15

**Authors:** E. G. Hasankhani, F. Omidi-Kashani

**Affiliations:** Orthopedic Department, Orthopedic Research Center, Emam Reza Hospital, Mashhad University of Medical Sciences, Mashhad 9186611111, Iran

## Abstract

*Introduction*. Radicular low back pain is one of the most common medical problems. The aim of this study was to evaluate the diagnostic accuracy of MRI and electrodiagnosis in lower extremity radicular pain in relation to history and clinical findings. *Methods*. In this cross-sectional study, we studied 165 sciatalgic subjects. A comprehensive history and physical examinations were taken from the subjects and recorded, and then MRI scanning and electrodiagnostic (nerve conduction velocity and electromyography) tests were performed. *Results*. From 152 subjects who remained in the study, 67 cases (44.1%) had radicular pain in left lower limb, 46 (30.3%) in right, and 39 (25.6%) in both lower limbs. 104 cases (68.4%) had shown some type of abnormalities in both MRI and electrodiagnosis, 30 (19.7%) had shown this abnormality only in MRI, and 21 (13.8%) only in electrodiagnosis, while 10 cases (6.5%) had both normal MRI and electrodiagnostic studies. Coordination rates of MRI and electrodiagnosis with clinical findings were 58.6% and 89.5%, respectively. *Conclusion*. In many MRI negative but symptomatic subjects, electrodiagnosis has an important diagnostic value.

## 1. Introduction

Radicular low back pain is one of the most common medical problems that cause decreased work competence and a heavy cost. Accurate diagnosis of this radicular pain has a paramount important role in proper treatment planning [1]. History taking and physical examination are the first steps in diagnosis of lower extremity radicular pain [2]. In clinical examination of these patients, in addition to the radicular pain, reduced muscle strength, a sensory deficit, and decreased deep tendon reflexes are reported [3].

The use of imaging techniques such as magnetic resonance imaging (MRI) is indicated in the patients with atypical or refractory complains to confirm the clinical diagnosis or help to select the proper approach if surgery is necessary [4].

Despite the accuracy of the history, physical examination and MRI in the lower extremity radicular pain, in some cases for more accurate diagnosis, other diagnostic measures are also needed. Although MRI has sufficient accuracy in the diagnosis of some nondiscogenic sciaticas such as spinal tumors, epidural varicosis, and infectious spinal stenosis, it is incapable of diagnosis in many far out (extraforaminal) spinal stenosis lesions. Electrodiagnostic tests can especially provide useful information about the exact location of the nerve damage [5].

Among all the electrodiagnostic studies, electromyography (EMG) technique has a very high accuracy and specificity in the diagnosis of nerve root pathologies such as denervation and dysfunction [6–8]. 

There is little research comparing the accuracy of MRI with electrodiagnostic methods in the diagnosis of lower extremity radicular pain; therefore, the aim of this study was to do this in relation to history and clinical findings.

## 2. Materials and Methods

At first, 165 patients with sciatica (±accompanying LBP) participated in the study. These subjects have been referred to our orthopedic department from November 2008 to December 2011. Our inclusion criteria were sciatica >6 weeks, age >15 years, and assignment of the informed consent, while we excluded those cases with a history of lumbar spine surgery, previous trauma, presence of associated disease (like Parkinson's disease, tuberculous spondylitis or brucellosis), underlying malignancy or autoimmune disease, and those patients that medically have contraindications for MRI or electrophysiologic studies. Eventually, 13 cases were excluded due to the exclusion criteria. The remaining 152 patients, 96 patients (63.2%) were males and the rest (56 cases; 36.8%) females. The mean age of the patients was 43 ± 5.8 (range from 22 to 73 years).

After a complete explanation of the project was given to the patients, they signed the informed consents. Demographic individual profile was recorded in a checklist. The history obtained from patients was about the nature of pain, period of pain, patient's occupation, and other symptoms that all were recorded in the individual checklist. Clinical examination included complete neurological evaluation was also recorded. All the patients had lumbosacral X-ray and MRI scanning that both were reported by an experienced radiologist. For electrodiagnostic study including both EMG and NCV, the patients were also referred to one expert physiatrist. For motor study, tibial, peroneal, and femoral nerves were evaluated while for sensory study, sural, saphenus, superficial peroneal, lateral, and posterior cutaneous nerves of thigh were checked. When the nerve root irritation was founded in both MRI and electrodiagnostic test, there was a concordance between MRI and electrodiagnostic findings. After collection of data forms, positive findings between clinics and paraclinics were compared and analyzed by software package for statistical analysis (SPSS, version 11), Chi-square, and independent *t*-tests.

## 3. Results

67 cases (44.1%) had radicular pain in left lower limb, 46 (30.3%) in right, and 39 (25.6%) in both lower limbs. Clinical and paraclinical findings in our patients were shown in Tables 1 and 2, respectively. Prevalence of abnormal findings in our paraclinical studies is as follows: 104 cases (68.4%) had shown some type of abnormalities in both MRI and electrodiagnosis, 30 (19.7%) had shown this abnormality only in MRI, 21 (13.8%) only in electrodiagnosis, while 10 cases (6.5%) had both normal MRI and electrodiagnostic studies. 

When the history and physical examination are taken into account, clinical accuracy of our paraclinical studies in lower extremity radicular pain is as shown in Table 3. Coordination rate (concordant) between MRI and the results obtained by the electrodiagnosis was 54%, while concordance of MRI and electrodiagnosis with clinical findings was 58.6% and 89.5%, respectively. For example in a paracentral L5-S1 disc herniation, it is obvious that imaging finding would not correlate with its clinical examination or nerve conduction studies. 

## 4. Discussion 

Our study compared MRI with electrodiagnosis and showed a high positive likelihood ratio for MRI, and therefore this method is considered a better modality to confirm the disease, while negative likelihood ratio for electrodiagnosis was high, or this method is a better one to roll out the disease. The high specificity of electrodiagnostic findings also has a clinical significance. Disc herniation in MRI scanning of the asymptomatic patients is a very common finding and therefore decision for surgery based on only MRI findings is not justified [9].

As our study showed in the patients with lower extremity radicular pain the high concordance of electrodiagnosis with final clinical diagnosis (89.5% relative to 58.6% in MRI scanning) indicated the high accuracy of this modality in these patients. In this study, we found that MRI has a less accuracy and more false positive in patients with canal stenosis and the use of electrodiagnosis is very effective especially in cases with multilevel canal stenosis to determine the location of pain. Our results supported the consequences of the study conducted by Johnsson et al. [10]. As Coster et al. [2] emphasized, electrodiagnosis cannot be replaced with MRI scanning. In the nondiscogenic sciaticas, the main etiology of the disease (like epidural varicosis, facet joint synovial cyst, etc.) cannot be found with this modality. 

There is not a gold standard method in the diagnosis of lower extremity radicular pain, and especially in deciding to select between surgical and nonsurgical planning, other methods in addition to history and physical examination are sometimes needed. Although, MRI scanning is a very popular method used to confirm the clinical diagnosis of radicular limb pain, in some cases, it is not suffice to decide the proper treatment planning [11].

In a study conducted by Pfirrman et al. (2004), they showed that MRI scanning has high accuracy in the diagnosis of discogenic radicular pain, but it is less accurate in the cases with nondiscogenic sciatica [12]. Patel and Lauerman in a separate study also found the same result [13]. In our research, the highest accuracy rate was found in the patients with disc herniation and spinal stenosis.

Our study showed that the accuracy of MRI scanning in the diagnosis of radicular limb pain (except in discogenic sciatica) is limited and to achieve a definitive diagnosis and treatment planning, other diagnostic methods are sometimes needed. Grover in a review confirmed this result [14]. In their study, when MRI scanning failed to be helpful in diagnosis and treatment planning, other paraclinical diagnostic methods such as electrodiagnosis have been used successfully. 

Although electrodiagnostic studies are not used as a routine procedure in diagnosis of lower extremity radiculopathies, they may be useful as a diagnostic aid in certain cases. These studies are useful in determining the relatively exact location and extent of nerve root involvement and they may be especially helpful in selecting appropriate treatment planning in MRI negative patients (cases with neuritis, diabetic neuropathy, and radiculopathy of an improved herniated disc). Clinically, neuropathic pain is sometimes too similar to the sciatic pain. To differentiate between the two, electrophysiologic study is very helpful [15]. Chiodo et al. (2007) found that needle electromyography is useful in differentiating symptomatic from asymptomatic disc herniation. They noted that this modality has a lower false positive rate than MRI in asymptomatic older patients that being evaluated for lower limb radicular pain [16]. 

In conclusion, although electrodiagnosis is not used as a routine and standard procedure in the diagnosis of lower extremity radiculopathy, in many MRI negative but symptomatic patients, this modality has an important diagnostic value. 

## Figures and Tables

**Table 1:** Positive clinical findings in our patients.

Positive sign	Number of cases (%)
Sensory impairment	134 (88.1)
Decreased deep tendon reflex	80 (52.6)
Motor impairment	79 (51.9)
Neurogenic claudication	24 (15.8)
Positive straight leg raising	47 (30.9)

**Table 2:** Paraclinical (MRI and electrodiagnosis) findings in our patients.

Paraclinical findings	Number of cases (%)
MRI	
(i) Disc herniation with nerve root compression	54 (35.5)
Level L4-L5	22 (14.5)
Level L5-S1	20 (13.2)
Level L4-L5 and L5-S1	12 (7.9)
(ii) Spinal stenosis	71 (46.7)
Level L3-L4	7 (4.6)
Level L4-L5	30 (19.7)
Level L5-S1	21 (13.8)
Multilevel	13 (8.6)
(iii) Degenerative disc without nerve root compression	15 (9.9)
(iv) Normal MRI scanning	12 (7.9)

Electrodiagnosis	
(i) Radiculopathy	91 (59.9)
(ii) Neuropathy	21 (13.8)
(iii) Both radiculopathy and neuropathy	13 (8.5)
(iv) Normal electrodiagnosis	27 (17.8)

**Table 3:** Clinical accuracy of MRI and electrodiagnosis in sciatica.

Paraclinical study	Sensitivity	Specificity	PLR^†^	NLR^x^	PPV^*⋆*^	NPV^#^
MRI	89%	11%	1	1	58%	39%
Electrodiagnosis	85%	39%	0.95	1.08	89%	87%

^†^PLR: Positive likelihood ratio.

^x^NLR: Negative likelihood ratio.

^*⋆*^PPV: Positive predictive value.

^#^NPV: Negative predictive value.
